# Survey of checkpoints along the pathway to diverse biomedical research faculty

**DOI:** 10.1371/journal.pone.0190606

**Published:** 2018-01-16

**Authors:** Lindsay C. Meyers, Abigail M. Brown, Liane Moneta-Koehler, Roger Chalkley

**Affiliations:** 1 Office for Biomedical Research Education and Training, School of Medicine, Vanderbilt University, Nashville, Tennessee, United States of America; 2 Department of Molecular Physiology and Biophysics, School of Medicine, Vanderbilt University, Nashville, Tennessee, United States of America; Universidade de Mogi das Cruzes, BRAZIL

## Abstract

There is a persistent shortage of underrepresented minority (URM) faculty who are involved in basic biomedical research at medical schools. We examined the entire training pathway of potential candidates to identify the points of greatest loss. Using a range of recent national data sources, including the National Science Foundation’s Survey of Earned Doctorates and Survey of Doctoral Recipients, we analyzed the demographics of the population of interest, specifically those from URM backgrounds with an interest in biomedical sciences. We examined the URM population from high school graduates through undergraduate, graduate, and postdoctoral training as well as the URM population in basic science tenure track faculty positions at medical schools. We find that URM and non-URM trainees are equally likely to transition into doctoral programs, to receive their doctoral degree, and to secure a postdoctoral position. However, the analysis reveals that the diversions from developing a faculty career are found primarily at two clearly identifiable places, specifically during undergraduate education and in transition from postdoctoral fellowship to tenure track faculty in the basic sciences at medical schools. We suggest focusing additional interventions on these two stages along the educational pathway.

## Introduction

Science benefits from diversity, and in this regard, there have been many reports that reflect a recognition of the inherent value of a more diverse workforce[1,2]. Indeed, based on numerous findings of social scientists, it is widely accepted that better discoveries and more creative solutions will emerge from an ethnically and racially diverse group of individuals working together. Yet, minorities remain seriously underrepresented in science and engineering[2]. Since minority groups are predicted to become the demographic majority in the United States by 2050, much effort has been devoted to increasing their representation in the scientific workforce[1]. To reach this goal, the National Institutes of Health (NIH) is committed to supporting institutions that are actively trying to recruit and retain more diverse trainees along the biomedical research educational pathway. As a result, we examined minority representation during all stages of the educational pathway to see where diversity efforts are most needed.

Individuals classified as underrepresented minority (URM) are scientists who self-identify with a racial or ethnic minority group “underrepresented in science” (see Methods section for full details). In Science, Technology, Engineering, and Mathematics (STEM) fields and medical schools, there is a shortage of URM faculty[3,4]. Within STEM, Howard Garrison examined the educational pathway in all STEM fields using a “synthetic cohort” method to measure the longitudinal career retention for those who expressed an interest in science or engineering upon matriculation into college[4]. He found that URM participants were leaving the pathway to faculty at all stages of training. The greatest losses were due to reduced undergraduate graduation rates[4,5].

Within the biomedical research community, some have noted a rising number of PhD candidates [6], though we find that over the last decade this increase is only seen for URM biomedical research trainees. We have explored how this growth in URM postgraduate trainees has affected the biomedical research workforce in general. While there has been increased representation of underrepresented groups in biomedical graduate education, those underrepresented groups are not showing up in tenure track faculty positions at levels that reflects either the demographics of the population at large or the number of URM trainees who have completed doctoral education[3]. A recent study by Gibbs et al focused on basic science medical school departments, and used simulations to understand the variables contributing to the lack of faculty diversity. They found that, based on current trends, merely increasing the number of URM doctoral awardees would fail to bring about sufficient or noticeable change in URM representation on faculty bodies. More importantly, it was found to be necessary in order to have an impact to change the rate at which postdoctoral fellows were hired into tenure track positions[7].

When constructing our study, we applied the “synthetic cohort “methods of Garrison to the more specific field of biomedical research because of the substantial investments made by the National Institutes of Health (NIH) to increase diversity in this area[4]. The synthetic cohort design (also known as a pseudo-longitudinal design) involves combining cross-sectional data from multiple time points to identify patterns over time. Unlike a true longitudinal study, each time point consists of different samples and, thus, includes more confounds than a “true” longitudinal study which follows the same individuals across the same timeline. For example, pseudo-longitudinal studies cannot account for differences across samples nor how the measurements may change over time. On the other hand, cross sectional data are much more easily obtained than “true” longitudinal data. In our analysis, we were able to use readily available data from the US Department of Education and National Science Foundation (NSF) which sample academic institutions on an ongoing basis. Using this method, we sought to determine where in the educational process the URM losses occur.

Thirty years ago it was accurate to state that URM trainees were being lost at every stage in the process. However, this analysis of currently available US national data sources indicates that the diversions of URM scholars from the goal of developing a faculty career specifically in biomedical research are found primarily at two highly identifiable places, (1) during undergraduate education, and (2) in transition from postdoctoral fellow to faculty. As a secondary question, we looked at the broader trends among biomedical education and how the field is growing. While there had been a large increase in biomedical PhD students from 2000–2008, after this date the numbers of matriculating and graduating doctoral students are no longer increasing.

## Methods

### Population

This study focused on the students and postdocs who contribute to diversity specifically in those areas of basic biomedical research that are substantially supported by grants from the NIH. URM trainees are defined as individuals who self-identified as belonging to one or more of the following racial or ethnic groups: Black or African-American, Hispanic or Latino, and American Indian or Alaskan Native. “Other” students refers to US citizens who have self-identified with two or more races or ethnic groups, or those who have chosen not to identify with any ethnic group, or those whose ethnicity is unknown to those conducting the survey. In the national data sources those who identified as Native Hawaiians or Pacific Islanders are grouped under the Asian American or Asian category which made it impossible to track them as a unique group underrepresented in science. Additionally, the appendix tables available from the NSF’s surveys did not report students with physical disabilities nor those who came from an economically disadvantaged or rural background. Therefore, we focused our study on those URM groups that have data consistently available across all national sources.

### Two analysis methods (synthetic cohort and historical)

We sought to identify the points of diversion in the pathway to tenure track URM faculty, which extends from graduating high school seniors to the stage of securing tenure track faculty positions in biomedical research. We used two methods to examine the pathway to faculty positions (synthetic cohort and historical data). The synthetic cohort method, similar to that used by Garrison et al[4], was used to examine the early stage educational pathway of a cohort of individuals who graduated from high school in 2009 and received a bachelor’s degree in biological sciences four years later in 2013. As described above, our use of the synthetic cohort design allows us to measure attrition on a national level on a very broad scale. The analysis does not look at the factors which contribute to attrition but rather focuses on the raw number of students who can be counted at certain checkpoints (high school graduation, college matriculation, and college graduation).

To study the demographics of the later stage educational pathway, we used available national historical data from 2000–2013. This second method examines the demographics of those following a traditional trainee trajectory from undergraduate, to graduate student, through postdoctoral fellowship to a tenure track faculty position over this time period. The data sources are clearly identified in S1 Table. In dissecting the data from these national sources, it is important to emphasize that we focused on very specific disciplines that tend to be most invested in basic biomedical research.

### High school graduation

The population of students graduating from public and private high school and the population of those recent high school graduates who matriculate into college (2 and 4 year institutions, full and part-time students) was obtained from the *Digest of Education Statistics* [8] and the *Private School Universe Survey* [9], both of which are supported by the National Center for Education Statistics (NCES). For public high school graduates, we used appendix tables which reported the number of graduates in each ethnic group in 2009 [8]. With respect to the number of private high school graduates by ethnicity, the appendix tables were more limited. In order to estimate this number of private high school graduates by ethnicity, we used the following two charts: 1) the number of private high school graduates in 2009 and 2) the private high school enrollment numbers by ethnicity in 2009 [9].

### College matriculation

To estimate the number of college matriculants in each ethnic group in 2009, we used data collected by the NCES, which provided the “percentage of recent high school completers enrolled in 2 and 4-year colleges [10].” Our cohort of interest most likely matriculated into college soon after high school rather later in life, leading us to choose “recent high school completers” population data. The data reported at this stage by the NCES does not include information on citizenship status.

### Early interest in biological sciences

At this point, we considered those first-year college students who have expressed an interest in biological sciences in the “Survey of the American Freshman [11].” In this survey, the field of biological sciences includes biology, biochemistry, biophysics, botany, environmental science, marine science, microbiology, bacteriology, zoology, agricultural sciences, and what is listed as ‘other’ biological sciences. The data tables released by the National Science Board report first-year college students’ interests by ethnicity and by field in the category of biological/agricultural sciences. This category is much broader than our final focus on biomedical research doctoral graduates; however, it is the only way to capture our targeted population of interest—those who will eventually earn an advanced degree in a biomedical research discipline. We estimated the number of freshmen who intend to major in biological or agricultural sciences in 2009 by using the estimated first-year population described above and the percentage of each ethnic group who express an interest in biological or agricultural sciences.

### College graduation with degree in biological sciences

The next checkpoint for our group of interest occurred four years later when we expected students to be graduating with a bachelor’s degree in the life sciences. The size of this cohort comes from a direct measure of degrees conferred in the biological sciences as reported in the National Science Board’s *Science and Engineering Indicators 2016* report [12]. From this point onwards we omitted those who end up majoring in agricultural sciences, a cohort which is consistently 20% of the overall size of the biological sciences degree recipients as this subset is now reported separately [12]. Note that in the denominator of the percent URM, we have included only US citizens and permanent residents throughout the study.

### Transition to examining historical data (2000–2013)

At this point in our study, we move from using the synthetic cohort method to examining the available demographic data from undergraduate bachelor’s degree receipt to faculty appointment from 2000–2013. In both methods (synthetic cohort for the early stage pathway and the historical data for the later stage pathway), we identified the demographics of those earning bachelor’s degrees in biological sciences [12].

### Graduate enrollment

Next, we examined the demographics of the doctoral population enrolled in biological sciences graduate programs. The *Science and Engineering Indicators 2016* appendix tables [13] do not distinguish between master’s and doctoral enrollment in these programs; therefore, we estimated the size of the doctoral population by subtracting the number of earned master’s degrees from the total enrollment in biological sciences graduate programs [13]. Assuming a typical master’s degree takes two years to complete, we excluded the number of earned master’s degrees in the current and subsequent year for 2002–2012 [13]. For 2013, we subtracted twice the number of master’s degrees conferred in 2013 to estimate the doctoral enrollment in that year as data on earned master’s degrees were unavailable for 2014. Again, when calculating percent URM, we included only US citizens and permanent residents in our denominator.

### Doctoral graduation

Graduation from a doctoral program is our next educational checkpoint. For the entire period 2000–2013, we used the NSF’s Survey of Earned Doctorates to examine the total number of degrees awarded in the broad field of biological sciences [14]. Fortunately, for the most recent time period of 2009–2015, we were also able to explore graduation data specifically from the sub-fields of basic science research programs in which we are particularly interested [15–21]. Prior to 2009, sub-field specific data were not available. For the years 2009–2015, we focused our analysis on the following biomedical basic sciences fields: Biochemistry, Bioinformatics, Biomedical sciences, Biophysics, Cancer biology, Cell/cellular biology and histology, Immunology, Microbiology, Molecular biology, Neurosciences, Pharmacology, Physiology, Toxicology, Biology, Anatomy/developmental biology, Bacteriology/parasitology, Endocrinology, Genetics, and Biotechnology. We excluded the fields of Botany, Epidemiology, Ecology, Entomology, Evolutionary Biology, Nursing Science, Nutrition Sciences, Zoology, and Wildlife Biology. Prior to 2009, the data charts available that show ethnicity composition do not provide sub-field level detail [15–21].

### Postdoctoral fellows

We next studied the size and diversity of the postdoctoral fellow population using information from the NSF *Survey of Doctorate Recipients* [22]. Due to a lack of sub-field-specific data available from the NSF, we were forced to use demographic data for those postdoctoral fellows in the life science fields who received a doctoral degree from a US institution.

### Faculty

Finally, we ended our examination of the pathway with an analysis of URM individuals in academic tenure track positions. We compared two data sources, the NSF and the American Association of Medical Colleges (AAMC) Faculty Roster. The NSF reports on the diversity of faculty by using the broad category of the life sciences population. However, the life science category is a very broad descriptor, and wherever possible, we have tried to focus on the basic biomedical research field. As 49 of the top 50 recipients of NIH funding (2014) for biomedical research are medical schools we have focused on data regarding URM faculty at these institutions [23]. Given our interest in NIH-funded diversity programs, we feel it is reasonable to focus on measures of faculty diversity in AAMC schools, even though medical schools are not the only institutions where basic biomedical scientists seek academic employment. The AAMC data on faculty diversity reflect demographics of faculty in tenure track positions involved in biomedical research in basic sciences departments, which more closely aligns to our cohort of interest [3].

## Results

### URM students express a strong interest in biological sciences upon matriculation into college

In 2009 in the US, 93.8% of Whites, 87.1% of African Americans, and 76.8% of Hispanics graduated from high school or equivalency in the age cohort of 18–24 year olds [24]. It is instructive to look at the timeline of the most recent cohort of students for whom we have college completion data. These students completed high school in 2009 and graduated with a bachelor’s degree in 2013. Of all URM students who entered a four-year college in 2009, between 8.6 and 11.0% (varies by demographic group) initially indicated they had an interest in biological sciences as described above (S1 Fig) [11]. This decision is comparable to the intentions of White students at 9.0%[11]. The numbers in 2009 reflect a trend which shows an increasing interest in biological sciences over the period of 1998–2014 for all ethnic groups [11].

Table 1 shows the educational progression of our synthetic cohort through undergraduate education. The first labeled column of Table 1 shows that White students made up 63.6% of the high school graduates in 2009, whereas URM students made up 30.9%. The second labeled column shows that URM students comprised 25.2% of the college enrollees who recently graduated from high school. The third labeled column indicates the size of the population of those freshmen who expressed an interest in the biological sciences. At this stage, URM students comprised 26.7% of all those who express an interest in this area. In the last column, we see that URM students made up only 17.8% of the students who earned a bachelor’s degree in biological sciences in comparison to White students who made up 59.7% of the population of earned bachelor’s degree in biological sciences in 2013. When examining those who earned an undergraduate degree in biological sciences, URM students comprised only 17.8% of the graduates, which is a significant decrease from the 26.7% of students who initially considered a focus in this area.

**Table 1:** Demographics of synthetic cohort along career pathway.

	Total private and public high school graduates (2009)*[8,9]	Estimated enrollment in 2 year and 4 year colleges by recent high school completers all institutions (2009)[10]	Estimated freshmen who express an interest in Biological/Agricultural Sciences (2009) [11]	Students who earn a bachelor’s degree in Biological Sciences (2013)[12]
	(% of all students)	(% of all students)	(% of all students)	(% of all students)
**All Students**	3,318,868 (100%)	2,609,769 (100%)	270,850 (100%)	101,663 (100%)
**White Students**	2,111,803 (63.6%)	1,480,374 (56.7%)	133,234 (49.2%)	60,732 (59.7%)
**URM Students**	1,026,923 (30.9%)	657,230 (25.2%)	72,295 (26.7%)	18,103 (17.8%)
**Other US Citizens Students**	not reported	314,721 (12.1%)	34,619 (12.8%)	6,787 (6.7%)
**Asian American Students**	180,142 (8.5%)	157,444 (6.0%)	30,702 (11.3%)	16,041 (15.8%)

*Please note that individuals may be counted twice if they identify themselves with multiple ethnicities.

### High attrition for URM students during undergraduate education

In contrast to Table 1 where we examined the demographics of the population at each stage in their undergraduate education, in Table 2, we compared the persistence of each ethnic group along this same early educational pathway. Each row shows the number and the percent of high school graduates who persisted to the next educational checkpoint. Row B shows that 70.1% of the White students who graduated from high school in 2009 choose to enroll in a two or four year institution in comparison to 64.0% of the URM students and 87.4% of the Asian American students. URM and White students express a similar interest in biological sciences (Row C). Row D shows that 2.9% of White students and 1.8% of URM students who graduated from the high school in 2009 completed a bachelor’s degree in biological sciences four years later in 2013. When we examined undergraduate degrees earned in 2013 (Row D), we see that for all groups the number of graduates is dramatically less than anticipated based on the number initially expressing an interest in biological sciences as first-year students in 2009 (Row C). The retention rate from expressed interest to completion of degree in biological sciences is 25.0% for URM students and 45.6% for White students (Row E). Here we see a major loss of URM biological scientists during undergraduate training.

**Table 2:** Population who persist to degree in biological sciences.

		White Students *(% of high school graduates)*	URM Students *(% of high school graduates)*	Asian American Students *(% of high school graduates)*
**A**.	Total private and public high school graduates (2009)	2,111,803 (100%)	1,026,923 (100%)	180,142 (100%)
**B**.	Estimated enrollment in 2 year and 4 year colleges by recent high school completers all institutions (2009)	1,480,374 (70.1%)	657,230 (64.0%)	157,444 (87.4%)
**C**.	Estimated freshmen who express an interest in Biological/Agricultural Sciences (2009)	133,234 (6.3%)	72,295 (7.0%)	30,702 (17.0%)
**D**.	Students who earn a bachelor’s degree in a Biological Sciences (2013)	60,732 (2.9%)	18,103 (1.8%)	16,041 (8.9%)
**E**.	Percentage of those who persist from interest to degree (Row D/C)	45.6%	25.0%	52.2%

Although we have used the graduation of our 2009–2013 synthetic cohort above as illustrative of a major leak in the pathway, this concern is not new as seen in S2 Fig where we show that this disparity among demographic groups has a long history with comparable losses since 2002.

As our investigation moves from using the synthetic cohort model to examining the historical population data from 2000–2013, we focus on the later educational pathway of our population of interest (biological sciences bachelor’s degree receipt to faculty position at a AAMC institution in a basic science department).

### Growth in bachelor’s degrees awarded to URM students in biological sciences (2000–2013)

**Fig 1:**
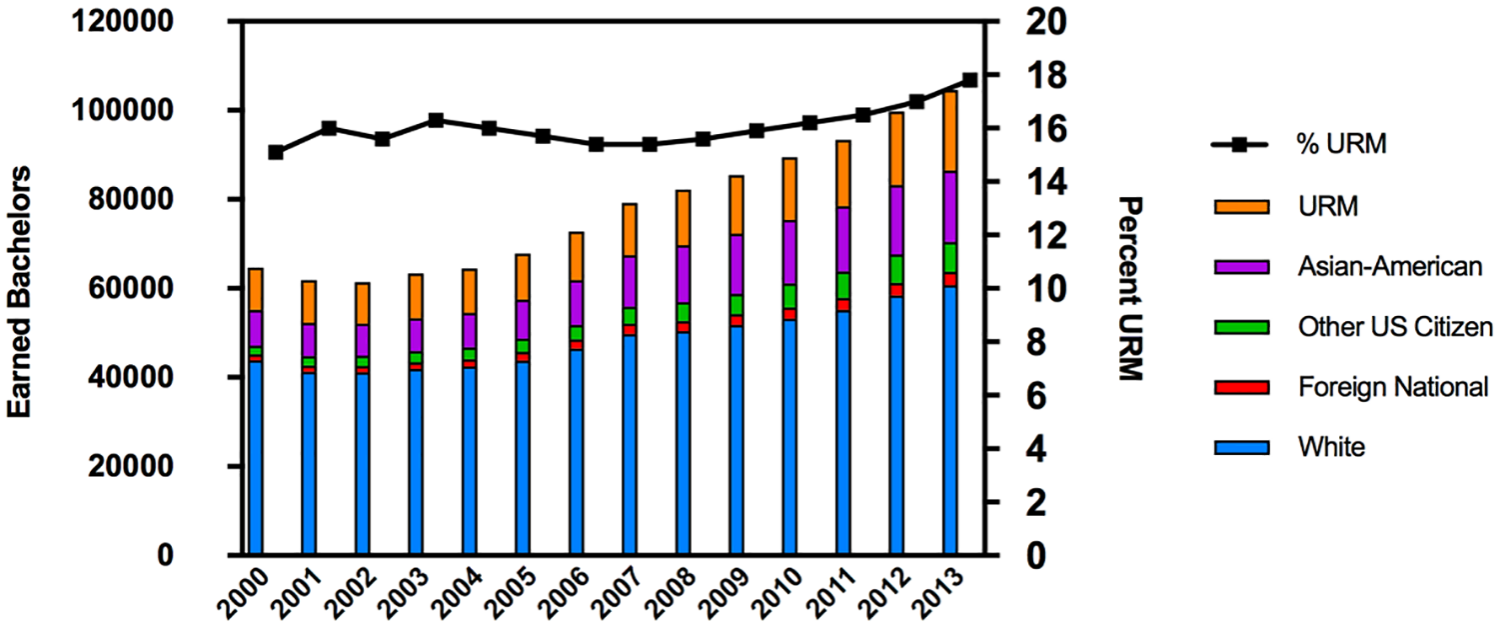
Increase in numbers of earned bachelor’s degrees in biological sciences by URM students. The left y-axis shows the number of earned bachelor’s degrees in biological sciences by ethnic group and the right y-axis shows the percentage of URM students who comprise the population of US citizen degree recipients in the field of biological sciences.

The distribution of earned bachelor’s of science (B.S.) degrees in biological sciences from 2000–2013 is shown in Fig 1. Over this time, the number of degrees awarded to URM students has doubled, in comparison to the number of degrees awarded to White students, which has increased by 38% [12]. By 2013, URM students comprised 18% of the US citizens who graduated with a degree in this discipline [12].

### URM enrollment in graduate programs in biological sciences (2000–2013)

**Fig 2:**
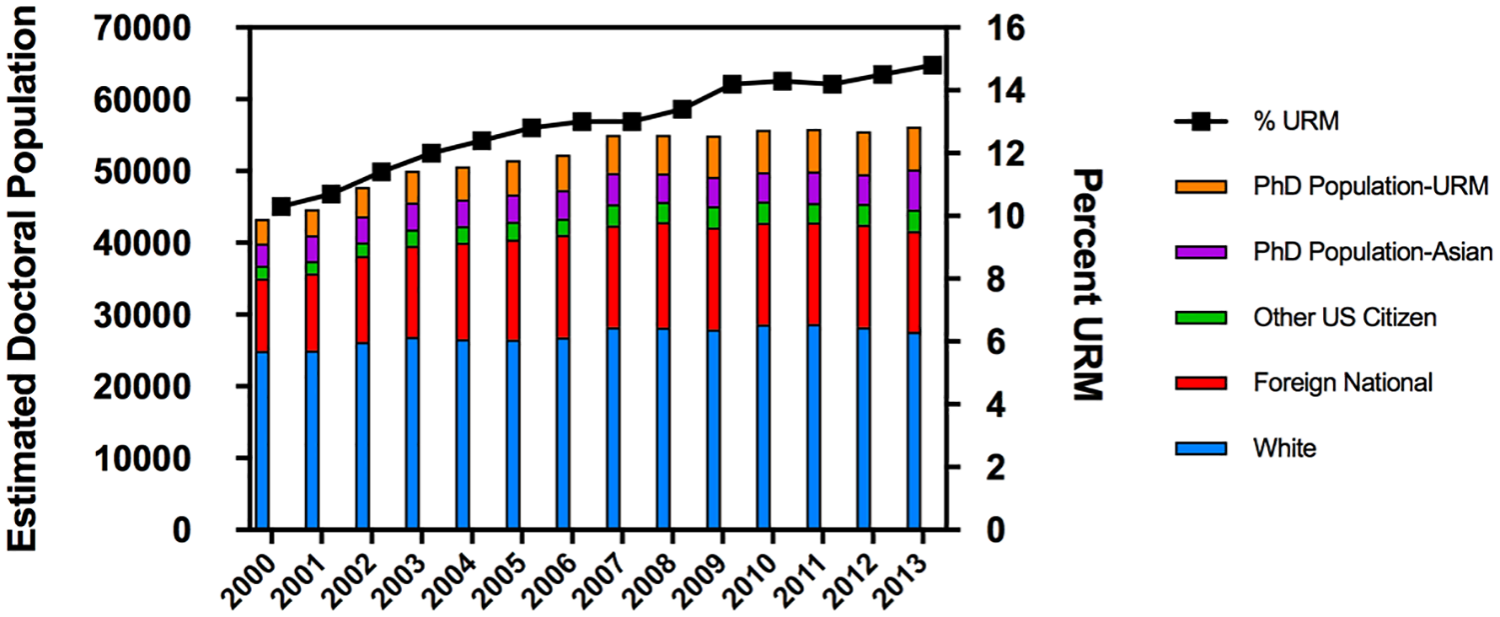
Calculated number of doctoral students enrolled in biological sciences graduate programs. The left y-axis shows the estimated total doctoral population enrolled in biological sciences graduate programs grouped by ethnicity and the right y-axis shows the percentage of the population comprised of URM students (US citizens in the denominator).

The estimated pre-doctoral enrollment in biological sciences graduate programs is shown in Fig 2, which covers the period 2000 to 2013 [13]. The source of most students for a graduate program is those undergraduates who earned a bachelor’s degree within the same discipline. We acknowledge that not all students entering a biological science doctoral program will have a bachelor’s degree in this same field; however, the vast majority of students do. At the national level, we wanted to compare the doctoral enrollment data in Fig 2 to the overall number of earned bachelor’s degrees in biological sciences in Fig 1. It is immediately apparent that the substantial increase in the total number of B.S. graduates, especially from 2004–2013, is not recapitulated in an increased enrollment in graduate education over this time period. More specifically, the total number of White, Asian, and international students in biological sciences doctoral programs is unchanged since 2007. In a remarkable contrast, the fraction of URM students entering graduate school has increased steadily since 2000 by nearly 74% (3,444 in 2000 to 5,992 in 2013).

A comparison of the number of URM students graduating with a BS in biological sciences from 2007–2013 (averaging 16–18% of biological science graduates) with the URM student population enrolled in graduate school (14–15% of the total) reveals that the number of URM students entering graduate school is largely determined by the fraction graduating with a bachelor’s degree in biological sciences. It is not clear how the number of URM students in graduate school could be significantly increased without decreasing the losses within undergraduate programs.

### Doctoral degrees awarded to URM students (2000–2013)

**Fig 3:**
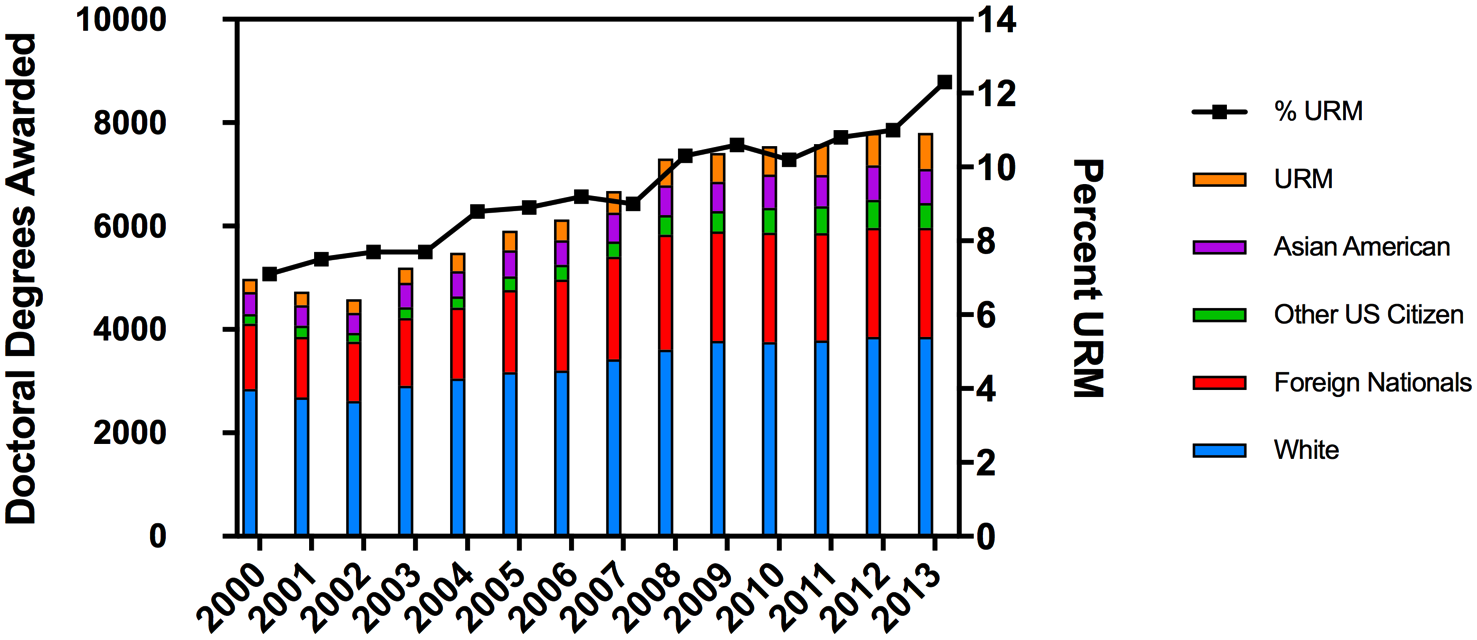
Earned doctorates in biological sciences programs. Number of awarded doctoral degrees and percent awarded to URM students. The left y-axis shows the number of earned doctorates in biological sciences by ethnicity and the right y-axis shows the percentage of the population composed of URM students.

As shown in Fig 3, from 2002 to 2008 there was an overall 64% increase in the number of doctoral degrees awarded which may be attributed in part to the doubling of the NIH budget [14]. Another driving factor was a doubling of the number of international students in biological sciences graduate programs over that period [14]. Quite remarkably, since 2008 the total number of doctoral degrees awarded annually to all students, except URM students, has remained constant. In contrast, the total number of degrees awarded to URM students has continued to grow. The figure shows a 2.7 fold increase in the number of URM PhDs awarded from 2000 to 2013 (263 in 2000 and 702 in 2013) [14].

**Fig 4:**
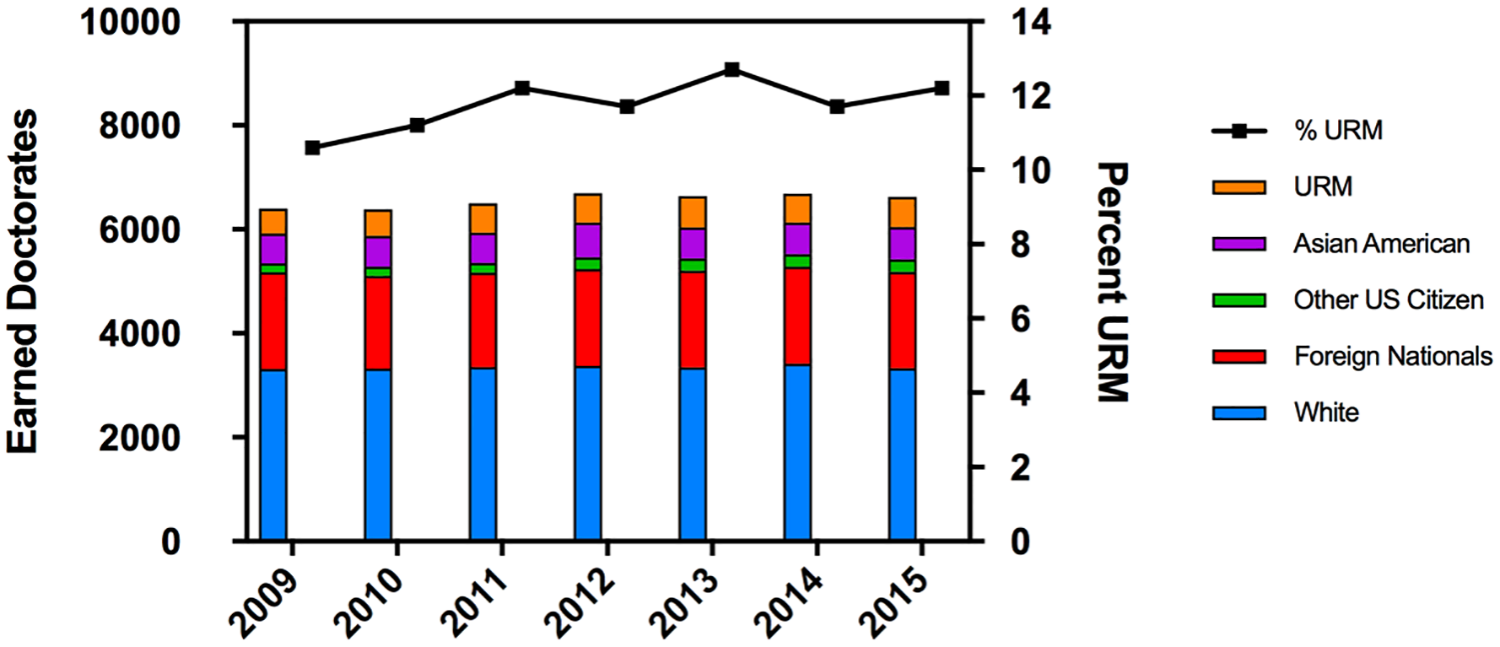
Earned doctorates in specific biomedical research fields. This includes degrees in Biochemistry, Bioinformatics, Biomedical sciences, Biophysics, Cancer biology, Cell/cellular biology and histology, Immunology, Microbiology, Molecular biology, Neurosciences, Pharmacology, Physiology, Toxicology, Biology, Anatomy/developmental biology, Bacteriology/parasitology, Endocrinology, Genetics, and Biotechnology [15–21].

Although Fig 3 provides some insight into the shifting demographics of doctoral awardees in the biological sciences from 2000 to 2013, we are most interested in the narrow cohort of doctoral recipients in the biomedical research fields as shown in Fig 4 [15–21]. The total number of doctoral degrees awarded in these specific disciplines since 2009 has held steady, but the proportion awarded to URM students has continued to increase to just above 12% of the total. Given that the number of URM students enrolled in biological sciences programs over this time ranged from 13.5–14.8% (Fig 2) we conclude that attrition of URM students from graduate programs was less than 15%, a number comparable to that of White students. Several points emerge: (1) the overall annual number of doctoral degrees awarded is no longer increasing, (2) the number of majority and international graduates is likewise at a plateau or slowly declining, (3) there is an increase for the smaller cohorts of Asian Americans and URM graduates, the latter increasing by about 15% from 2009 to 2015 (482 URM graduates in 2009 compared to 585 URM graduates in 2015).

### Involvement of URM doctoral recipients in postdoctoral training (2001–2013)

**Fig 5:**
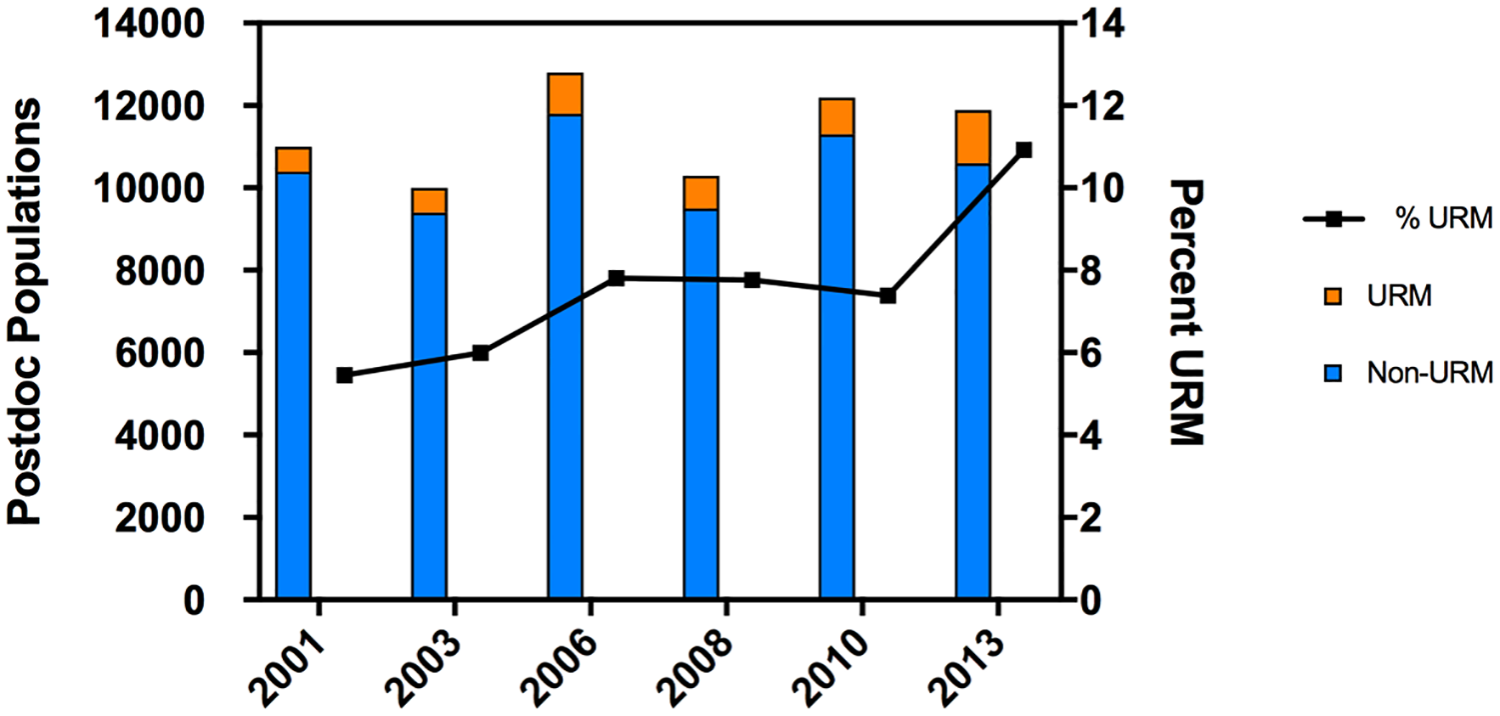
Postdoctoral population in life sciences at academic institutions. This reflects the biennial *Survey of Doctorate Recipients*. The left y-axis shows the postdoctoral population employed in academic institutions who received degrees from a US institution and who are employed at academic institutions. The right y-axis shows the percentage of the population comprised of URM fellows.

The next step in the journey to becoming a faculty member almost always requires securing a postdoctoral fellowship at an academic institution. With increased URM enrollment in and graduation from biomedical research programs, we examined demographics of the postdoctoral fellowship population that earned a doctoral degree in the US. Again, due to the lack of specific information available, we had to return to the broadly defined “life science” fields (Fig 5) [22]. In 2015 there were nearly 12,000 academic postdoctoral fellows in the life sciences who earned a doctoral degree from a US institution. The number of US non-URM postdoctoral fellows in these fields, while somewhat variable, has not increased in the last 15 years. In contrast, the relative fraction of URM postdoctoral fellows has doubled in the last 12 years. By 2013, 12% of doctoral awards were earned by URM trainees, and URM graduates made up 11% of the postdoctoral fellows who received their doctoral degree from a US institution. This argues that the newly minted URM PhDs secure a postdoctoral position at a similar frequency to White doctoral awardees. Because the data collection could include some international PhD recipients who earned their doctoral degrees in the US, this 12% URM representation should be viewed as a minimal estimate.

### Reduced transition of URMs into faculty positions (2001–2013)

As reported by the NSF, the number of URM full-time faculty in the life sciences has increased from 2,000 in 1993 (4% of all faculty) to 5,200 in 2013 (7.3%) [22]. We are unable to determine which of the 5,200 are in tenure track positions nor can we determine the type of institution employing the individuals (liberal arts, non-research intensive, and research intensive); however, roughly 22%, or 1,000 primarily African-American, life sciences faculty are at Historically Black Colleges and Universities (HBCUs) [25]. The NSF numbers are encouraging but do not represent our population of interest—those employed in tenure track positions at AAMC basic science departments. In 2013, URM basic science faculty made up around 6% of those appointed to assistant or associate professor positions and only 4% of those appointed to the rank of full professor [3]. Despite the steady increase in URM participation in the biomedical research postdoctoral community, an examination of the current tenure track faculty rosters at AAMC institutions shows that individuals from minority groups are still highly underrepresented relative to their presence in graduate school and postdoctoral programs.

**Fig 6:**
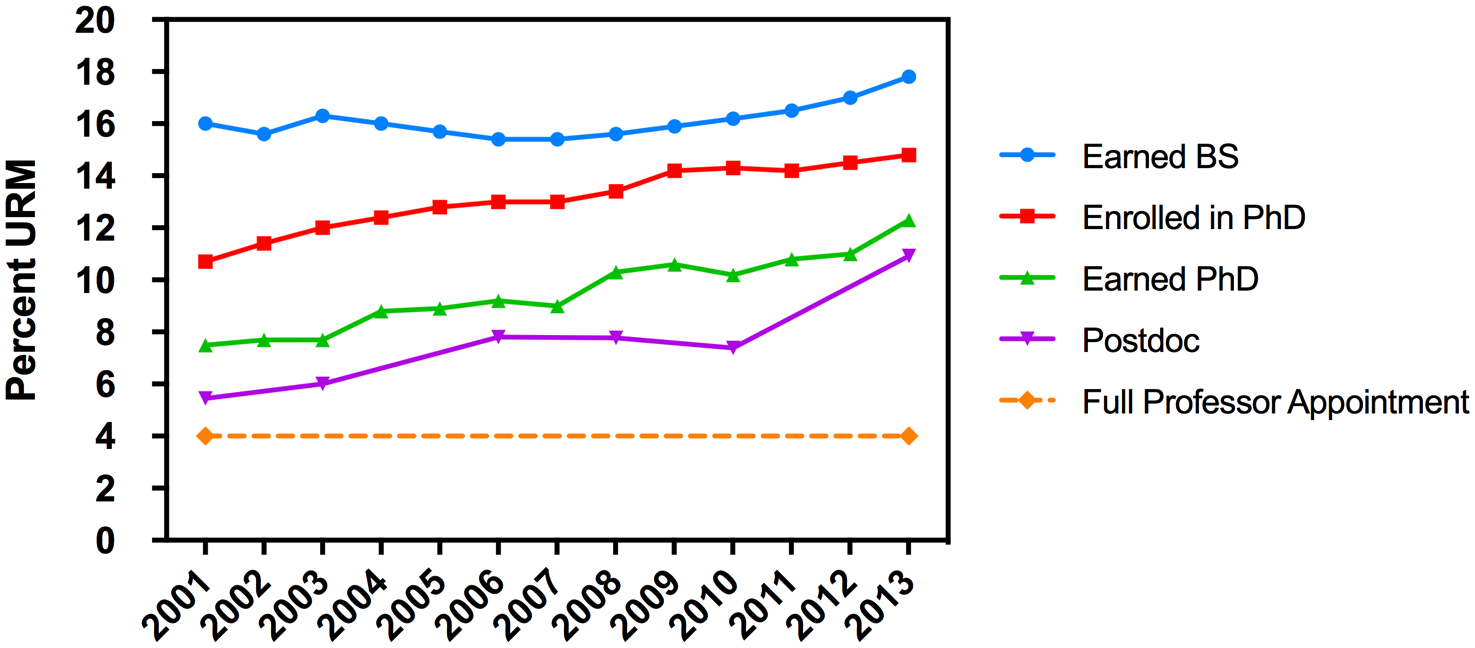
Percentage of URM trainees at critical checkpoints prior to faculty appointment. The Y-axis shows percentage of URM within the following populations: Earned Bachelor’s Degree in Biological Sciences, Doctoral Enrollees in Biological Sciences Graduate Programs, Doctoral Graduates in Biological Sciences, Postdoctoral Fellows in Life Sciences, and Full Professors AAMC basic science departments.

A summary of the increasing levels of participation by URM students and postdoctoral fellows in the academic environment leading to tenure track faculty positions is shown in Fig 6. The figure shows increasing participation in (a) graduation with an undergraduate degree in biological sciences [12] (b) matriculation into biomedical graduate programs[13], (c) graduation with a doctoral degree[14], and (d) training as a postdoctoral fellow [22]. In all instances the trend lines show a steady and continuing increase. Participation in graduate school by URM trainees is up significantly and doctoral degree receipt is up by almost 50% from 2000 to 2013 [14]. Participation by URM postdoctoral fellows has doubled during this same time period [22]. Despite these measured increases, there is no comparable increase in the number of URM trainees who advance to tenure track basic science appointments in AAMC medical schools. Although there are obviously a variety of other academic institutions outside AAMC schools where individuals will be involved in biomedical research, we believe medical school basic science departments are a useful proxy to measure the URM faculty participation overall in biomedical sciences.

## Discussion

In this paper we have examined the various checkpoints where URM individuals are lost from the pathway leading to faculty positions. This analysis reveals there are two times that persist as areas of concern, in addition to three checkpoints where minimal losses occur. We discuss these various checkpoints in order of their occurrence.

The first major interruption in the pathway to faculty is during the stage of undergraduate education. Over the last fifteen years there has been an increasing number of degrees awarded to URM students, which is clearly an accomplishment; however, the graduation rate is below expectations based upon the number of incoming undergraduate URM students who express an interest in graduating with a biological sciences degree. This poor retention rate likely reflects nation-wide attrition from college, which has been shown to be higher among minority groups and economically disadvantaged students [5,26].

Similar to our findings, the NCES released a report that found that attrition rates in non-STEM fields were as high or higher than those in STEM fields in their defined cohort. Additionally, their study found that nearly one-half of the students in their STEM cohort did not complete a degree in STEM [5,26]. Six years after matriculation, NCES found that White STEM students were dropping out of college or switching to a non-STEM major at a rate of nearly 50% in comparison to URM students who were leaving college or STEM fields at a rate of 50–70%, which is consistent with the data that we have found specific to the field of biological sciences across all institutions over a 4-year period. These conclusions for biological sciences are in general agreement with observations reported for other STEM programs by Garrison [4]. In the NCES analysis, the researchers examined the following factors related to attrition: sex, race, education level of parents, family income level, highest math taken in high school, high school GPA and selectivity of undergraduate institution. While we acknowledge all of these factors are important to study, the purpose of our own national cohort analysis remains to examine the raw numbers of matriculants and graduates at each stage to identify the stages of greatest talent loss in the pathway to developing a diverse biomedical research faculty body.

Although it is possible to increase college completion rates by looking at a cohort of students six years after matriculation rather than four years, the relative outcomes of the different populations of students are not widely different enough to justify moving our analysis to examining our cohort at 6 years past matriculation into a bachelor’s degree. Both the NCES’s longitudinal cohort and our broad synthetic cohort all show that URM undergraduate attrition is an area of great loss of human capital to our field of interest in biological sciences, as well as to every other STEM and non-STEM field. Because this study is pseudo-longitudinal and does not follow the same sample of people, limitations exist. The cross-sectional data do not capture students who matriculate into an undergraduate or graduate institution after being out of school for an extended amount of time or students who take a leave of absence during their education. The data do not account for individual differences, such as individuals who may have participated in intervention programs targeted for underrepresented minorities. Despite these limitations, the national data available to us strongly demonstrate that URM undergraduate attrition is a major diversion of candidates from a trajectory leading ultimately to faculty positions. There is a critical need for interventions focused on improving retention of all students at the undergraduate level.

After the significant loss of URM students during undergraduate studies, our study reveals three checkpoints of minimal loss in the pathway to faculty. These checkpoints are matriculation into a doctoral program, receipt of a doctoral degree, and acquisition of a postdoctoral position. Considering that URM students are earning roughly 18% of the bachelor's degrees in biological sciences in 2013, we are encouraged that 15% of the doctoral students and 13% of the doctoral graduates are URM. These strong rates of persistence are promising to those who have worked hard to design interventions targeting persistence from graduate school matriculation to graduation. Similar to doctoral graduation persistence, we see comparable URM retention at the postdoctoral level. In 2013, URMs made up 11% of the postdoctoral fellows employed at academic institutions in the life sciences which also demonstrates that URMs are interested in pursing a postdoctoral fellowships at a similar rate to White doctoral graduates. Clearly, overall, one of the major positive outcomes detected by this study is the observation that the fraction of URM trainees is steadily increasing at every stage in the analysis over the entire 13 years (2000–2013).

We credit several successful interventions for reinforcing the integrity of the educational pathway for a URM trainee. An almost certain factor in this trend is the success of a range of programs devised by the NIH and NSF, such as the MARC, PREP, IMSD, minority summer programs, and especially the T32 programs at the NIH. By requiring intentional recruitment of and participation from diverse trainees on all T32 grants, the NIH plays a major role in increasing diversity at the graduate and postdoctoral level. A recent National Research Council study showed that an institution’s minority student population increases in an environment supported by NIH training grants [27].

Although minority participation increased in graduate training and in doctoral receipt from 2007–2013, the overall student and graduate numbers (all students) in the biomedical sciences field remained constant. This is an extremely important observation as there is a popular misperception that the number of PhDs awarded in biomedical science graduate programs is increasing vigorously. In a recent article the statement was made that for the life sciences, “the number of doctoral recipients shows no signs of leveling off” [6]. While it is true that the number of degrees awarded to those in life science fields has continued to grow slowly, the number of doctoral degrees awarded to those in the traditional and basic biomedical research fields has not grown while minority participation continues to increase. These data reveal that URM students are just as successful at matriculating into graduate school, completing their doctoral degrees, and securing a postdoctoral position as all other groups.

The second major interruption in the pathway to faculty in the basic sciences occurs immediately after a trainee’s postdoctoral fellowship. Compared to their non-URM peers, far fewer URM postdoctoral trainees transition into faculty positions in AAMC basic science departments, which is in agreement with a recent report from Gibbs et al on all STEM fields [7]. This large decline could be attributed to at least two factors. First, it is possible URM fellows make a specific and intentional choice not to apply for academic faculty positions due to a negative perception of the career. Gibbs et al. suggest that institutions will need to consider making “faculty positions and work environments attractive and supportive to these scientists, ensuring the proper types of support (e.g. funding, mentorship, and sponsorship) to allow URM postdocs to effectively progress to independence [7].” Because we lack national data, such as exit interviews, to determine the rationale for their career choices, we are left to speculate if URM trainees find a career in academic medicine unappealing or perceive the environment at an AAMC institution as inhospitable. Second, although it is possible that URM interest has shifted away from tenure track academic positions at AAMC institutions, it is also possible that URM trainees are less successful at securing a position even if their interest and experience in this career is as strong as any non-URM candidate. Again, we lack national data sources on applicant demographics from faculty searches that might shed light on this issue.

Ideally, one would like the demographics of the faculty in biomedical sciences to closely match those of the US population, which would result in at least a 30% URM composition. The NIH strives for more diversity within its scientific community believing that better ideas and discoveries will come from diverse groups [1,28]. However, the fact is that only 3–7% of biomedical research faculty positions are occupied by URM scientists depending upon the type of school. In a narrow examination of AAMC institutions, URM individuals make up between 3–4% of the medical school basic science full-time faculty appointed to the rank of professor [3]. In fact, over the course of the last fifty years, the percentage of URM professors in the basic sciences in medical schools has only increased from 2 to 4% [3]. Additionally, it is important to note that although there are around 700 URM basic science faculty employed at all medical schools, these individuals are not necessarily uniformly distributed across institutions. It is more likely that URM faculty are found in more concentrated numbers at a few HBCUs, such as Morehouse School of Medicine, Howard University College of Medicine, and Meharry Medical College.

In conclusion, within the last decade we see that minimal losses occurred during the transition into graduate school doctoral programs, attainment of a doctoral degree, and when securing a postdoctoral position. These are encouraging results; however, we remain profoundly concerned that URM trainees divert substantially from a biomedical faculty career during (1) undergraduate education and (2) in the final transition from postdoctoral fellowship to tenure track faculty position. Thus, as NIH, universities, and other institutions are committed to increasing diversity at the faculty level, they will need to focus their attention on the contributing factors to stages where major losses occur.

## Supporting information

S1 Fig Percent of first-year college students who express an interest in biological or agricultural sciences. For all ethnic groups, there is an increasing amount of interest in the biological sciences upon matriculation into college. Black, Latino, and White students express similar interest in this area.
(TIFF)

S2 Fig Percentage of students who complete the bachelor’s degree in biological sciences after expressing an interest in the area at matriculation. As seen in the synthetic cohort, Whites who express an interest in biological sciences are nearly twice as likely to complete a bachelor’s degree in biological sciences four years later than URM students.
(TIFF)

S1 Table Summary of data sources used for tracking educational pathways. (PDF)
