# Supplementary figures and images for: Survey of checkpoints along the pathway to diverse biomedical research faculty
Source: PLoS One. 2018 Jan 16;13(1):e0190606. doi: 10.1371/journal.pone.0190606 (PMC5770033; doi:10.1371/journal.pone.0190606)

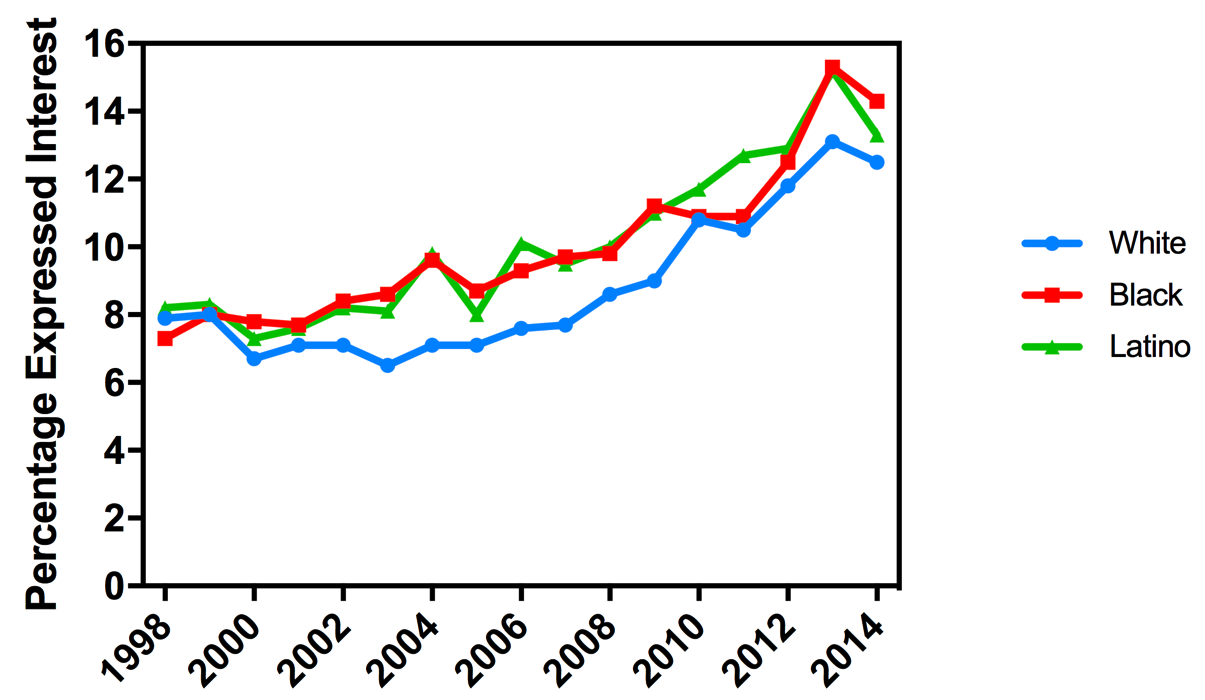

Supplement: S1 Fig — For all ethnic groups, there is an increasing amount of interest in the biological sciences upon matriculation into college. Black, Latino, and White students express similar interest in this area. (TIFF) [file pone.0190606.s001.tiff]

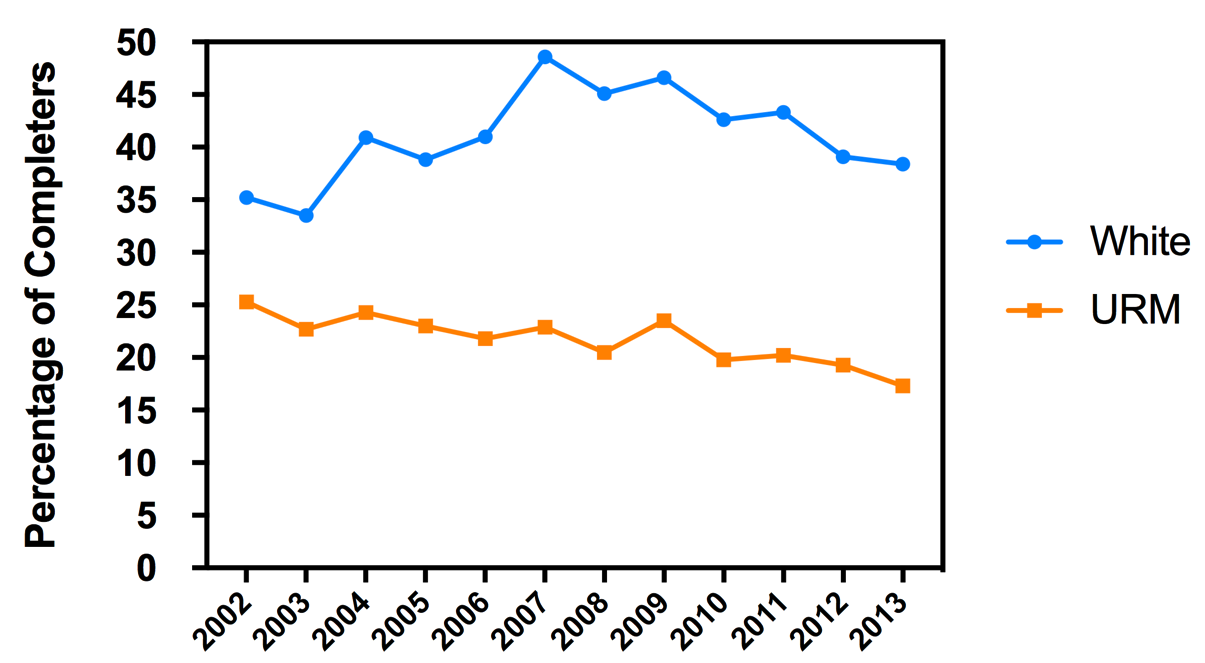

Supplement: S2 Fig — As seen in the synthetic cohort, Whites who express an interest in biological sciences are nearly twice as likely to complete a bachelor’s degree in biological sciences four years later than URM students. (TIFF) [file pone.0190606.s002.tiff]
