# Supplementary material for: Survey of checkpoints along the pathway to diverse biomedical research faculty
Source: PLoS One. 2018 Jan 16;13(1):e0190606. doi: 10.1371/journal.pone.0190606 (PMC5770033; doi:10.1371/journal.pone.0190606)
Supplement: S1 Table — (PDF) [file pone.0190606.s003.pdf]

## Supplemental Table 1. Summary of Data Sources Used for Tracking Educational Pathways

| Check point | Cohort of Interest | Source | Source limitations | Source Details | Associated Figures and Charts |
|---|---|---|---|---|---|
| Public and private high school graduates in 2009 | All graduates from public and private high schools | Digest of Education Statistics and Private School Universe Survey | The data available to us does not count 'other US citizens' in high school graduation data. We estimated the number of private school graduates of each ethnic group based on minority demographics at all schools and the total number of private high school graduates in 2009. | Digest of Education Statistics, 2011 Tables and Figures, Table 114, (https://nces.ed.gov/programs/digest/d11/tables/dt11_114.asp), Private School Universe Survey high school data, https://nces.ed.gov/surveys/pss/tables0910.asp (Table 13 and Table 9) | Chart 1a and Chart 1b |
| **Estimated** enrollment in 2 year and 4 year colleges by recent high school completers all institutions (in 2009) | Estimated college matriculant size based on the number of high school graduates and the demographics of the recent high school completers enrolled in 2 and 4 year colleges in 2009. | U.S. Department of Education, National Center for Education Statistics | | U.S. Department of Education, National Center for Education Statistics, Common Core of Data (CCD), "NCES Common Core of Data State Dropout and Completion Data File,"School Year 2009–10, Table 2 and U.S. Department of Education, Digest of Education Statistics, Table 302.20 "Percentage of recent high school completers enrolled in 2- and 4-year colleges, by race/ethnicity: 1960 through 2014" (https://nces.ed.gov/programs/digest/d15/tables/dt15_302.20.asp) | Chart 1a and Chart 1b |
| Estimated freshmen who intend to major in Biological/Agricultural Sciences (2009) | Calculated population based size of entering class from above and percentage of those intending to major in biological or agricultural sciences | National Science Board, Science and Engineering Indicators (2014) | Weighted by population for the URM (black, American Indian, and Hispanic). Assume 10.4% of those who are Other US citizens. | https://www.nsf.gov/statistics/2016/nsb20161/#/data , Appendix Table 2-16 Original source: Higher Education Research Institute, University of California at Los Angeles, Survey of the American Freshman: National Norms, special tabulations (2013). Weighted by population for the URM (Black, Hispanic, and American Indian) | Chart 1a, Chart 1b, Supplemental Figure 2, and Supplemental Figure 3 |
| Students who earn a bachelors in a Biological Sciences (2013) | B.S. in Biological Sciences | National Science Board's Science and Engineering Indicators 2016. | These numbers do not include those who completed a bachelors degree in agricultural science. We are aware of this unequal comparison within the cohort. | National Science Board's Science and Engineering Indicators 2016. https://www.nsf.gov/statistics/2016/nsb20161/#/data, Appendix Table 2-18 | Chart 1a, Chart 1b, Figure 1, Supplemental Figure 3 |

| Career Stage | Cohort of Interest | Data Source | Method for Focused Cohort | Source Details | Associated Figures and Charts |
|---|---|---|---|---|---|
| Population of earned bachelors degrees in biological sciences | Biological sciences bachelor's degree earners | NSF (Survey of Earned Doctorates) | Direct from appendix table | Science & Engineering Indicators 2016. https://www.nsf.gov/statistics/2016/nsb20161/#/data, Appendix Table 2-18. | Chart 1a, Chart 1b, Figure 1, Figure 6 |

| | | | | | |
|---|---|---|---|---|---|
| Estimated number of doctoral students enrolled in biological sciences programs | Biological sciences doctoral students | NSF (Survey of Earned Doctorates) | Subtracting the biological sciences masters degrees earned in the current year and subsequent year from the total graduate enrollment in graduate programs in biological sciences. | Science & Engineering Indicators 2016. https://www.nsf.gov/statistics/2016/nsb20161/#/data, Appendix tables 2-26 and 2-29 | Figure 2, Figure 6 |
| Earned doctorates in biological sciences programs | Biological sciences doctoral recipients | NSF (Survey of Earned Doctorates) | Direct from appendix table | Science & Engineering Indicators 2016. https://www.nsf.gov/statistics/2016/nsb20161/#/data, Appendix tables 2-30 | Figure 3, Figure 6 |
| Earned doctorates in biomedical research fields | Doctoral recipients from basic science biomedical research fields | NSF (Survey of Earned Doctorates) | Subtracted the graduates from the fields of Botany, Epidemiology, Ecology, Biostatistics, Entomology, Evolutionary biology, Nutrition sciences, and Zoology | National Science Foundation, National Center for Science and Engineering Statistics, Survey of Earned Doctorates (2009, App. Tab. 20), (2010, App. Tab. 22), (2011, App. Tab. 22), (2013. App. Tab. 22), (2014. App. Tab. 22), (2015. App. Tab. 22) | Figure 4, Figure 6 |
| Postdocs in Life Sciences | Postdoctoral fellows who completed a doctorate at a US institution who are now working in a academic institution in the Life Sciences | NSF (Survey of Doctoral Recipients) | Direct from appendix table | National Science Board. Science & Engineering Indicators 2016. National Science Foundation. Washington, DC. https://www.nsf.gov/statistics/2016/nsb20161/#/data, Appendix Table 5-15. | Figure 5, Figure 6 |
